# Microstructural evolution and mechanical characterization of a WC-reinforced CoCrFeNi HEA matrix composite

**DOI:** 10.1038/s41598-022-13649-5

**Published:** 2022-06-14

**Authors:** Syed Waqas Hussain, M. Adil Mehmood, M. Ramzan Abdul Karim, Andy Godfrey, Khurram Yaqoob

**Affiliations:** 1 School of Chemical and Materials Engineering (SCME), National University of Sciences and Technology (NUST), H-12, Islamabad, Pakistan; 2 Department of Materials Science and Engineering, Ghulam Ishaq Khan Institute of Engineering Sciences and Technology, Topi, Swabi, 23640 KPK Pakistan; 3 Laboratory of Advanced Materials (MoE), School of Materials Science and Engineering, Tsinghua University, Beijing, 100084 China

**Keywords:** Mechanical engineering, Materials science

## Abstract

High entropy alloys (HEAs) are a relatively new class of material that have shown the potential to exhibit excellent combinations of mechanical properties. Various microstructural modifications have been explored to further enhance their mechanical properties for use in demanding structural applications. The main focus of the present work is an investigation of the effect of adding varying amounts of hard ceramic material (WC) to a tough HEA matrix (CoCrFeNi) by arc melting under an argon atmosphere, including microstructural changes, and evaluation of the WC additions on mechanical properties. X-ray diffraction analysis of the HEA-WC composites showed the presence of both fcc and carbide phases. Scanning electron microscope investigations, including energy dispersive spectroscopy, reveal that chromium diffuses from the matrix and interacts with WC to form an alloyed carbide phase. The amount of alloyed carbide was found to increase with increasing amount of WC addition to the HEA matrix. Mechanical characterization revealed that hardness and yield strength of the HEA-WC composites increase with increasing amount of the carbide phase in the matrix. The hardness of HEA-20wt.% WC sample was found to be as high as 3.3 times (593 HV) the hardness of the base HEA (180 HV), while the yield strength increased from 278 MPa for the base HEA to 1098 MPa for the CoCrFeNi-20 wt.% WC composite. The investigated composites also showed excellent values of ductility (~ 50% strain for CoCrFeNi-10 wt% WC and ~ 20% strain for CoCrFeNi-20 wt% WC). It is therefore believed that ceramic-reinforced high entropy matrix composites have the potential to provide outstanding combinations of mechanical properties for demanding structural applications.

## Introduction

Strength and toughness are the two key properties required in structural materials for safe endurance of high loads. Increases in strength, however, are in many materials inevitably accompanied by some sacrifice in ductility, with a corresponding loss of toughness. Grain refinement of coarse-grained Ni metal to the nanoscale, for example, has been reported to lead to an increase in yield strength from just 53 MPa to 1.3 GPa, but at the cost of a severe decrease in ductility (to < 5%)^1^. A number of different attempts have been made to overcome this strength-ductility trade off. Some notable approaches that have been tried in this regard include development of heterogeneous nanostructures^2^ or hierarchical microstructures^3,4^, use of nano-precipitation strengthening^5^, austempering^6^, and spheroidization^7^, as well as alloy design to encourage either transformation induced plasticity (TRIP)^8^ or twinning induced plasticity (TWIP)^9,10^. Nevertheless, the strength- ductility tradeoff remains an outstanding issue and it increasingly seems that the mechanical properties of conventional alloys are approaching their capacity limits.

High entropy alloys (HEAs) form a relatively new class of material that are based on the simultaneous presence of four or five or more elements in equal amounts^11,12^. HEAs are characterized by the presence of four characteristic effects, namely configurational entropy, severe lattice distortion, sluggish diffusion and the cocktail effect^11,13,14^. These effects are considered to be responsible for better combinations of properties in comparison to conventional alloys, including outstanding thermal stability, corrosion resistance, and fatigue strength, as well as superplastic elongation and better mechanical properties even at cryogenic temperatures^12,15–26^. HEAs have also shown the potential to exhibit enhanced combinations of strength and ductility. Excellent ductility can be achieved in fcc HEAs, albeit with limited strength, whereas high strength but limited ductility has been reported for bcc HEAs Different attempts have been carried out, therefore, to further enhance the strength-ductility combinations in HEAs by design of HEAs containing both fcc and bcc solid-solution phases, where some promising results have already been achieved^27–29^. Development of HEAs strengthened by interstitial solid-solutions has also been explored. Addition of nitrogen^30^, oxygen^31^ and carbon^32,33^ atoms into a HEA matrix has been reported to improve strength, but at the expense of limited ductility. HEAs utilizing the TRIP effect have also been developed in a quest for better combinations of mechanical properties^8,34,35^. Transformation of metastable phases under stress has in some cases been reported to improve the ability to resist fracture, however at present only a few systems have been shown to exhibit good results in this regard^8,34^. Development of a thin deformed layer and an undeformed core, connected by a gradient hierarchical microstructure^36^ in a HEA was also shown to improve ductility, but with only a small corresponding increase in strength. Additionally, eutectic HEAs, consisting of fine lamellae of hard and soft phases^37,38^, have also been developed by employing different alloy design strategies. The use of such eutectic microstructures in HEAs has shown promising results. Design and control of eutectic microstructures in HEAs is, however, an extremely difficult task, due to the simultaneous presence of multiple elements.

The idea of reinforcing a HEA matrix with a ceramic phase in order to obtain better combinations of mechanical properties has also recently emerged. Development of such HEA composites has been tried both by ex-situ additions of discrete ceramic particles and also by in-situ formation of ceramic phases in an HEA matrix^39^. The main focus in the case of HEA composites based on a fcc matrix is to improve the yield and tensile strength without compromising ductility, based on the idea that a fcc matrix may provide good toughness while the ceramic phase contributes to high strength. In a recent study, a CoCrFeMnNi fcc HEA was reinforced with TiC and TiN particles, resulting in a significant improvement in mechanical properties^40–42^. Similarly, in-situ formation of TiC has also been explored in the (CoCrFeNi)Al_x_Cu_y_ HEA system^43^. Improvement in the plasticity and fracture toughness of bcc HEAs may also help towards the goal of obtaining better combinations of strength and ductility. For example, addition of TiC and TaC to the Mo_0.5_NbHf_0.5_ZrTi bcc HEA^44,45^ resulted in the development of a eutectic microstructure and the presence of metal carbide precipitates, with the bcc phase in the microstructure helped to obtain high strength combined with good ductility.

In the present study we focus on development of ceramic-reinforced HEA composites with a fcc matrix as part of a wider search of materials with improved combinations of strength and ductility. The CoCrFeNi system, as one of the most widely studied fcc HEAs, was selected as the matrix material for the study. For the ceramic phase WC is chosen, due to is relatively good toughness and its widespread use as a high density ceramic material in demanding applications. Using these starting materials CoCrFeNi-WC composites were prepared by an arc melting process. The evolution of microstructure and crystal structure were studied as a function of WC weight fraction, together with an investigation of the mechanical properties of the HEA-ceramic composites.

## Experimental procedure

High purity elemental powders of cobalt, chromium, iron, and nickel, together with tungsten carbide (WC) ceramic powder (purity > 99.95%) were used as raw materials. Metal powders with varying additions of WC powder (0, 5, 10, or 20 wt%) were mixed in an agate mortar, followed by arc melting under a high-purity argon atmosphere to produce HEA composite buttons of 20 g weight. Each composition was re-melted five times, with samples flipped over after each melting to ensure chemical homogeneity. Measurements taken before and after melting showed a weight difference of less than 0.5%. Sectioning of the HEA composite buttons for characterization of mechanical properties, as well as for investigation of the phase composition, microstructure and chemical analysis, was carried out using electrical discharge wire cutting. Samples for microstructural evaluation were carefully prepared using standard metallographic preparation procedures and then etched with aqua regia. The microstructure was examined both using an optical microscope (OPTIKA-600) and a scanning electron microscope (SEM; JEOL JSM-6490LA and VEGA-3), equipped with a Bruker energy-dispersive X-ray spectroscopy (EDS) system. Crystal structure characterization of the composites was carried out using an X-ray diffraction (XRD) system, operated with a CuKα source at a step size of 0.04°. Vickers microhardness measurements were performed on polished cross-sectional surfaces using a 136° Vickers diamond pyramid indenter. The Vickers hardness (HV_0.3_) was measured under 300 N force using a 15 s dwell time. Room temperature compression testing was performed using a SHIMADZU universal testing machine with tests carried out at an initial strain rate of 1 × 10^–3^ s^−1^.

### Compliance with ethical standards

This study was funded by the Higher Education Commission of Pakistan (HEC-NRPU Project # 6019).

## Results and discussion

**Figure 1:**
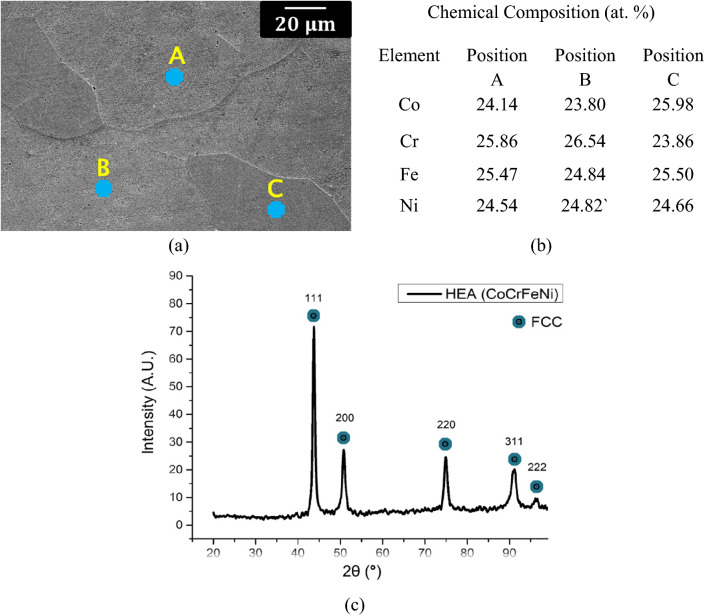
Characterization of the base CoCrFeNi HEA: (**a**) SEM micrograph, (**b**) EDS analysis of the three regions labelled in (**a**), and (**c**) XRD pattern showing the single fcc phase composition.

A sample of HEA matrix without WC addition was characterized prior to investigation of the HEA-ceramic composites to provide a reference material. An example SEM micrograph of the as-cast CoCrFeNi HEA is shown in Fig. 1a, together with accompanying EDS analysis at selected locations in Fig. 1b. The similarity of the EDS results in different locations confirms the chemical homogeneity of the single phase HEA. The XRD pattern of the base CoCrFeNi HEA, shown in Fig. 1c, also confirms that the base alloy was a single-phase fcc solid solution, in good agreement with the SEM microstructural observations.

**Figure 2:**
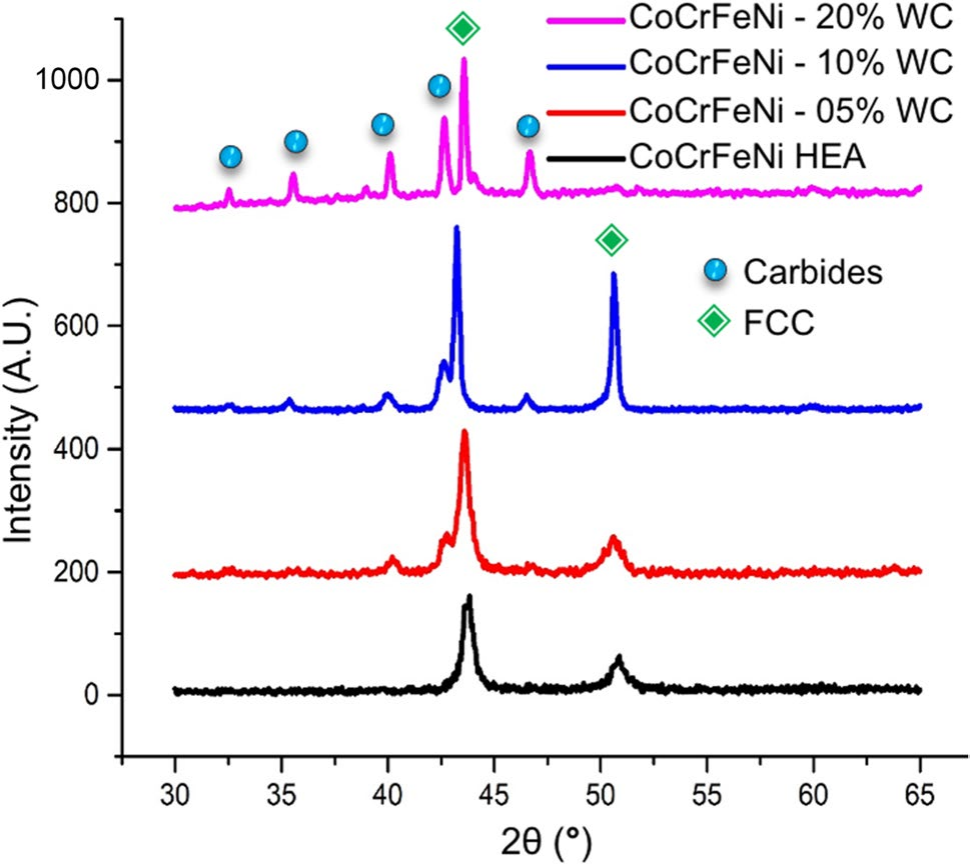
X-ray diffraction patterns of the CoCrFeNi-WC composites together with data for the single phase CoCrFeNi HEA.

**Figure 3:**
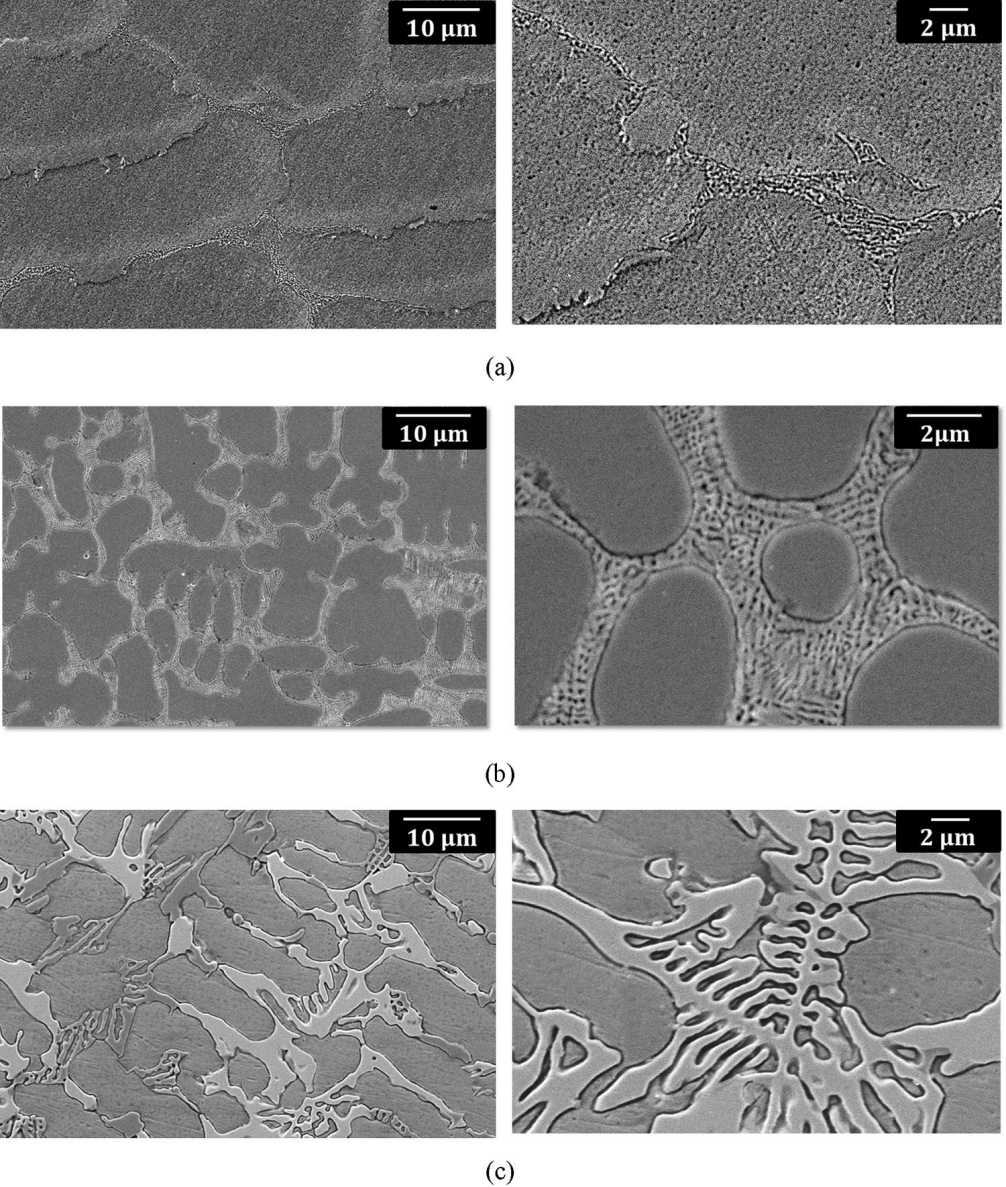
Example scanning electron micrographs of composite samples prepared from (**a**) CoCrFeNi-5 wt% WC (**b**) CoCrFeNi-10 wt% WC (**c**) CoCrFeNi-20 wt% WC. A eutectic microstructure consisting of an alloyed carbide phase (white) and a matrix phase (grey) can be seen, with an obvious increase in the eutectic phase volume fraction with increasing WC addition.

XRD patterns of HEA composites prepared by arc melting under argon atmosphere using 5, 10 and 20 wt% of WC are shown in Fig. 2, where the data for the single phase HEA over the same 2θ range are also reproduced for reference. The XRD data clearly show the presence of peaks corresponding to the fcc phase, as well from an alloyed carbide phase. No peaks corresponding to the presence of the pure WC were observed. The chemical affinity of chromium for carbon is much higher than the affinity of W for carbon (the Gibbs free energies of Cr_23_C_6_, Cr_7_C_3_ and Cr_3_C_2_ are − 343.9 kJ/mol, − 144.4 kJ/mol, − 72.3 kJ/mol, respectively^46^). It is therefore assumed that chemical interaction of WC and chromium takes place during melting, resulting in disintegration of the WC and formation of chromium carbides. Scanning electron microscopy and EDS analysis were carried out to further investigate this hypothesis and to evaluate the effect of WC additions on the microstructure of the CoCrFeNi HEA phase. Example SEM images of the high entropy composites with initial WC contents of 5, 10, and 20 wt% are shown in Fig. 3.

The SEM observations revealed the presence of a two phase microstructure, seen in the micrographs of Fig. 3 as grey matrix phase and lighter alloyed carbide phase (referred to in the following as white, though depending on SEM conditions this can also appear as light grey). The presence of a eutectic microstructure (consisting of a dark grey phase and white phase) is also seen at grain boundaries in the HEA-5wt%WC alloy (Fig. 3a). The amount of the eutectic phase was found to increase with increasing amount of WC addition.

**Figure 4:**
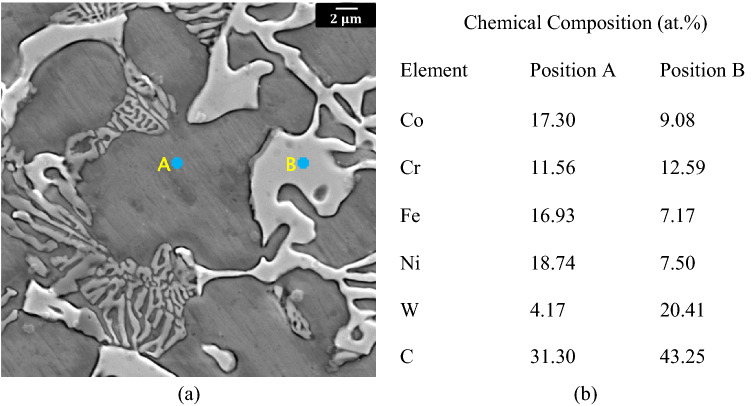
(**a**) SEM image and (**b**) EDS analysis of the CoCrFeNi-20 wt% WC composite.

Example chemical compositions of both the matrix and carbide regions in the CoCrFeNi-20 wt% WC alloy, as determined from EDS measurements, are shown in Fig. 4a,b. EDS analysis of the matrix (typified by position A in Fig. 4a) revealed more or less equiatomic concentrations of Co, Fe, and Ni, with a significant decrease in the amount of chromium as compared to the reference alloy (without WC addition). A small amount of W was also found in the matrix. Chemical analysis of the phase with white contrast (e.g., position B in Fig. 4a) showed relatively high amounts of Cr and W, consistent with diffusion of chromium from the matrix and chemical interaction with WC. This white phase was identified as an alloyed carbide, on the basis that the relative intensity of the peaks corresponding to an alloyed carbide phase in the XRD patterns also increased with increasing amount of WC addition in the HEA-ceramic composite.

Based on these observations it is believed that during melting interaction between WC and the HEA components, especially Cr, takes place, leading to the formation of an alloyed carbide phase. As a result the W becomes partially dissolved in the matrix and partially dissolved in the carbide phase. A comparison of the SEM images shown in Fig. 3 also reveals that addition of WC leads to a grain size refinement, both in the case of the 5 wt% WC composite and also for the higher wt% composites, as a result of the formation of the eutectic and alloy carbide phases. The volume fraction of the alloy carbide phase increases with increasing amount of WC addition, with a corresponding decrease in grain size A slight shift of the fcc peaks in the HEA-WC composites towards the left for the 5 wt% WC and 10 wt% WC alloys (in comparison to the base HEA), despite the outward diffusion of chromium, is explained by the fact that W, with large atomic size, becomes dissolved in the matrix. For the 20 wt% WC alloy a large amount of carbide phase is formed. As a result the effect on lattice parameter of outward diffusion of chromium from the fcc matrix dominates over that from addition of W to the matrix, resulting in a slight shift of the fcc peak to the right.

**Figure 5:**
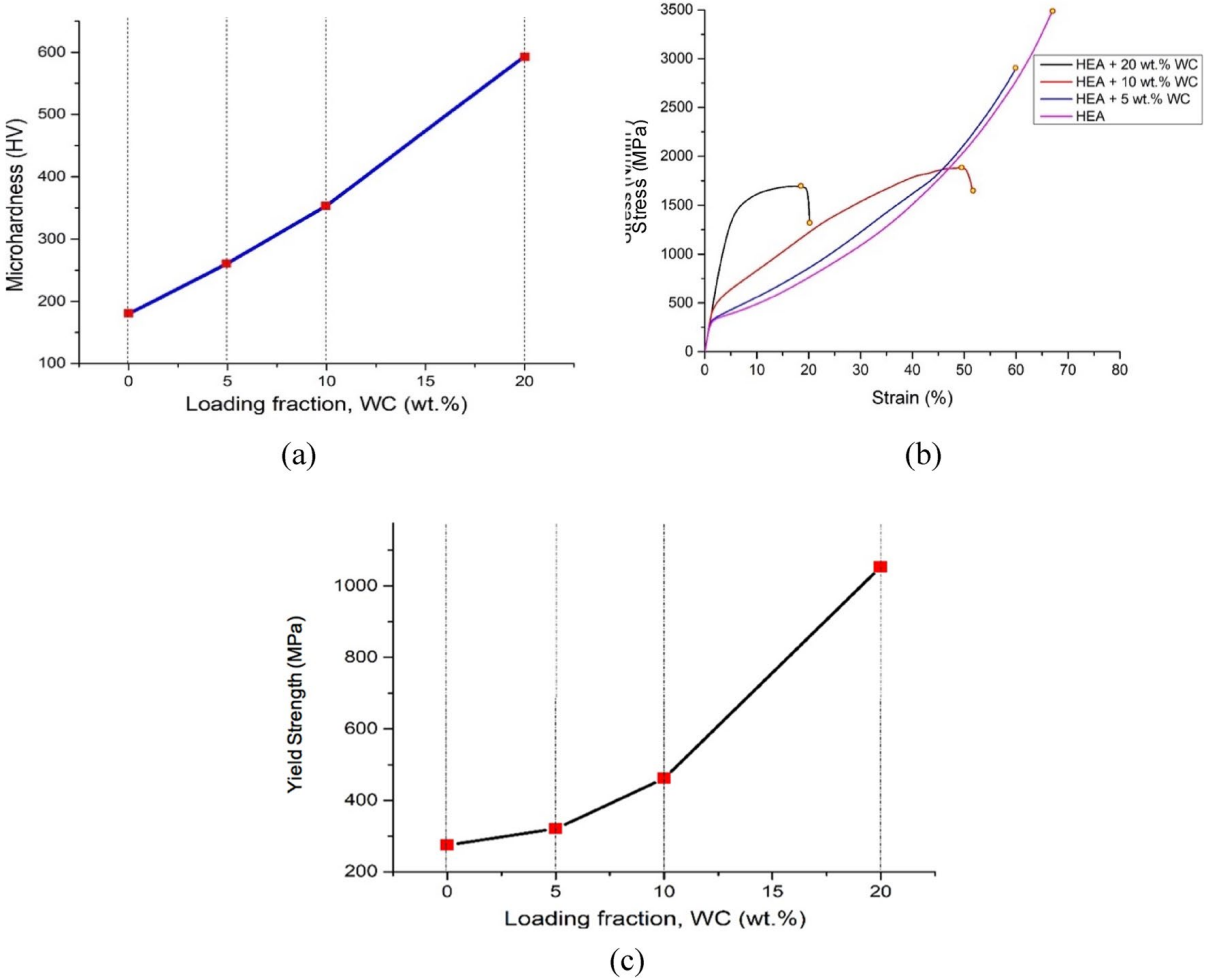
Mechanical properties of the HEA–WC composites: (**a**) variation of hardness as a function of amount of added WC (loading fraction); (**b**) compressive stress–strain curves; and (**c**) variation of yield strength (0.2% proof stress) as a function of amount of added WC.

The effect of the aforementioned microstructural changes on the mechanical properties of the HEA composites was evaluated by use of hardness and compression testing. The results of the hardness measurements are shown in Fig. 5a. The hardness of the base HEA was found to be 180 HV, increasing linearly with increasing amount of WC addition. The hardness of the HEA-20 wt% WC sample was found to be as high as 3.3 times (593 HV) the hardness of the base HEA. The increase in hardness was attributed both to lattice distortion resulting from diffusion of W into the matrix, as well as to the increase in the amount of the alloy carbide phase. The yield strength and ductility of the HEA-WC composites were evaluated by compression testing. Engineering stress–strain curves of HEA composites are shown in Fig. 5b, while the variation of yield strength (defined as the 0.2% proof stress) as a function of the amount of added WC in the master alloy is shown in Fig. 5c.

Compression test curves for the CoCrFeNi HEA and CoCrFeNi-5 wt% WC composites show a concave appearance, while compression curves of the CoCrFeNi-10 wt% WC composite and CoCrFeNi-20 wt% WC composite exhibit a convex shape. A stress–strain with concave appearance is typically characteristic of the activation of either twinning or TRIP effects, while dislocation glide-dominated deformation results typically in a convex profile^47,48^. The observations therefore suggest a change in the deformation mode as the amount of added WC in the base HEA (master alloy) increases to 10% and above. The variation of yield strength as a function of amount of WC in the HEA composite is shown in Fig. 5c. The yield strength increases from 278 MPa for the base HEA to 1098 MPa for the CoCrFeNi-20 wt% WC composite, mirroring the increase observed from the hardness measurements. In addition to the significant increase in the yield strength observed, the composites were found also to retain excellent values of ductility. In particular the HEAs with 10 wt% WC and 20 wt% WC addition exhibited good elongation before fracture, reaching values of about ~ 50% and ~ 20% strain, respectively, along with high values of compressive strength. An increase in strength resulting from the WC additions without significant decrease in ductility was attributed to three different aspects of the microstructural changes in the HEA composites: (1) grain size refinement, (2) dispersion strengthening, and (3) the presence of a fine eutectic microstructure consisting of hard (carbides) and soft (fcc) phases.

Grain size refinement is attributed to the promotion of heterogenous nucleation during solidification by chromium carbides, formed as a result of disintegration of WC and interaction of the released C with Cr in the HEA matrix. A fine grain size is known to help with slip homogenization, promoting ductility, and also leads directly to strengthening via the Hall–Petch relationship. Enhanced work hardening in the alloy due to twinning also extends the necking stage, thereby enhancing ductility^29^. Alloyed carbide particles are expected also be present in the form of fine particles dispersed in the matrix phase. The presence of such fine carbides can be expected to lead to an increase in work-hardening capability through dislocation-particle interactions, as well as to promotion of slip homogeneity within each grain. The latter results in a delayed build-up of dislocation pile-up stresses at the coarse grain boundary carbides, contributing also to the improved balance of strength and ductility in the composites. Similarly the presence of a mesoscale distribution of the larger alloy carbides (as seen in Figs. 3 and 4) can be expected to promote an increase during deformation in the density of geometrically necessary dislocations in regions of the HEA matrix adjacent to the harder alloy carbides. A difference in the both the thermal expansion coefficients and the elastic modulii between the HEA matrix and precipitated carbides has also been previously reported to enhance composite properties^40,49^.

The development of a fine-scale eutectic microstructure, consisting of a hard (carbide) phase and a softer fcc phase, also directly contributes to an improvement in strength. Moreover, the micrometer-scale distribution of this eutectic phase, in particular in the 10% WC composite sample, results in a hierarchical hard/soft microstructure, benefitting both the strength and plasticity of the HEA-WC composites. It can be noted also that the presence of the eutectic phase can also help to reduce castability problems, by reducing the possibility for deleterious segregation.

The results of the present study show that ceramic-reinforced high entropy matrix composites can help to achieve the goal of attaining alloys with improved combinations of strength and ductility. The selection of the ceramic reinforcement for a particular HEA matrix, and of the accompanying processing route is, however, very important in this regard. Among the elements present in the CoCrFeNi matrix, chromium has the strongest affinity for the formation of carbides, and therefore results in disintegration of WC due to the negative enthalpy for Cr carbide formation^46,50^. In a previous study the same HEA matrix was reinforced with WC by spark plasma sintering of mechanically alloyed HEA/WC powder. In that work the presence of four different phases, namely a fcc matrix phase, WC, M_23_C_6_-type carbides and M_7_C_3_-type carbides, was found^50^. The strength of the as-sintered composites was high, but the composite suffered from low ductility, as a result of the formation of the various carbides. This was attributed to the presence of a process control agent as well as other contamination during mechanical alloying. In another study, the CoCrFeNi HEA system was reinforced with WC and applied as a coating on a steel substrate by vacuum hot-press sintering, with the goal of improving the surface properties of the steel. The resulting microstructure contained some WC as well as other carbides, all of which were uniformly distributed in the fcc phase^51^, and an improvement in both hardness and wear resistance was found. Taken together with the present study, these results indicate that the same combination of ceramic reinforcement phase and HEA system can lead to the formation of different types of microstructure depending on the applied processing route, resulting in a modification of the mechanical properties to a different extent in each case.

## Conclusions

- Ceramic reinforced HEA-matrix composites have been successfully prepared by addition of WC to a CoCrFeNi matrix through an arc melting route.
- Chemical analysis and crystal structure analysis reveal interaction of WC with the HEA components. Specifically, chromium combines with decomposed WC to form an alloyed carbide phase in the fcc HEA matrix.
- Microstructural investigations reveal the presence of alloyed carbides both at the grain boundaries and as part of a eutectic phase. The amount of alloyed carbide phase was found to increase with increasing amount of WC addition in the HEA composite.
- Mechanical characterization reveals that the hardness and yield strength of the HEA composites increase with increasing amount of carbide phase in the matrix. The hardness of the HEA-20 wt% WC composite sample (593 HV) was found to be as high as 3.3 times that of the base HEA (180 HV), while the yield strength increased from 278 MPa for the base HEA to 1098 MPa for the CoCrFeNi-20 wt% WC composite.
- The HEA composites retain excellent ductility to failure (~ 50% strain for CoCrFeNi-10 wt% WC and ~ 20% strain for CoCrFeNi-20 wt% WC). It is therefore believed that development of HEA composites can help in achieving alloys with improved combinations of strength, ductility and toughness.

## Data Availability

The data used to support the findings of this study are included within the article.
